# Chronic subdural electrocorticography in nonhuman primates by an implantable wireless device for brain-machine interfaces

**DOI:** 10.3389/fnins.2023.1260675

**Published:** 2023-09-28

**Authors:** Tianfang Yan, Katsuyoshi Suzuki, Seiji Kameda, Masashi Maeda, Takuma Mihara, Masayuki Hirata

**Affiliations:** ^1^Department of Neurological Diagnosis and Restoration, Osaka University Graduate School of Medicine, Suita, Japan; ^2^Ogino Memorial Laboratory, Nihon Kohden Corporation, Tokyo, Japan; ^3^Candidate Discovery Science Labs, Astellas Pharma Inc., Tokyo, Japan; ^4^Department of Neurosurgery, Osaka University Graduate School of Medicine, Suita, Japan; ^5^Global Center for Medical Engineering and Informatics, Osaka University, Suita, Japan

**Keywords:** brain-machine interface, implantable device, electrocorticography, chronic tissue reaction, recording quality

## Abstract

**Background:**

Subdural electrocorticography (ECoG) signals have been proposed as a stable, good-quality source for brain-machine interfaces (BMIs), with a higher spatial and temporal resolution than electroencephalography (EEG). However, long-term implantation may lead to chronic inflammatory reactions and connective tissue encapsulation, resulting in a decline in signal recording quality. However, no study has reported the effects of the surrounding tissue on signal recording and device functionality thus far.

**Methods:**

In this study, we implanted a wireless recording device with a customized 32-electrode-ECoG array subdurally in two nonhuman primates for 15 months. We evaluated the neural activities recorded from and wirelessly transmitted to the devices and the chronic tissue reactions around the electrodes. In addition, we measured the gain factor of the newly formed ventral fibrous tissue *in vivo*.

**Results:**

Time-frequency analyses of the acute and chronic phases showed similar signal features. The average root mean square voltage and power spectral density showed relatively stable signal quality after chronic implantation. Histological examination revealed thickening of the reactive tissue around the electrode array; however, no evident inflammation in the cortex. From gain factor analysis, we found that tissue proliferation under electrodes reduced the amplitude power of signals.

**Conclusion:**

This study suggests that subdural ECoG may provide chronic signal recordings for future clinical applications and neuroscience research. This study also highlights the need to reduce proliferation of reactive tissue ventral to the electrodes to enhance long-term stability.

## 1. Introduction

Electrocorticography (ECoG) is widely used to accurately record neural signals, with electrodes placed either epidurally or subdurally. Thus, we can measure the signal amplitude at the millivolt (mV) level, which is significantly higher than scalp electroencephalography (EEG), and is less vulnerable to artifacts (Haynes and Rees, 2006; Baars and Gage, 2010). Unlike intracortical microneedle electrodes, ECoG electrodes do not penetrate the cortical tissue, avoiding blood–brain barrier damage, thereby potentially mitigating the inflammatory response and extending its functional duration (Campbell and Wu, 2018; Yan et al., 2020). Clinically, ECoG has been used to diagnose epileptogenic zones in presurgical monitoring since the 1940s (Nakasato et al., 1994). Nowadays, there is growing interest in using chronic ECoG electrodes in brain-machine interface (BMI, also known as brain-computer interface) applications to control neuro-prosthetic limbs or synthesize speech from neural activity in paralyzed patients (Bouchard et al., 2013; Vansteensel et al., 2016; Anumanchipalli et al., 2019; Benabid et al., 2019; Miller et al., 2020).

In contrast to clinical use, in which electrodes are generally implanted for no more than 30 days for epilepsy monitoring, for BMI applications, it is crucial to ensure long-term safety and stable functionality to deliver high-quality neurophysiological data. An ideal device requires good biocompatibility, high selectivity, low invasiveness, and a long working period (Alahi et al., 2021). Several studies have shown that ECoG recordings can record high gamma frequency (from 90 to 200 Hz) activity with reliable performance over multiple years (Chao et al., 2010; Ryapolova-Webb et al., 2014; Nurse et al., 2018; Larzabal et al., 2021). We previously observed minimal tissue reactions of the subdural electrode array after a 6-month-implantation in beagles (Yan et al., 2020). Some longer-term studies have also shown a stereotypical foreign body response with inflammatory cell accumulation and connective tissue proliferation at the tissue-array interface on both the dorsal (dura mater side) and ventral (arachnoid/brain tissue side; Degenhart et al., 2016). Despite the studies on evaluating the host-tissue response of implanted electrodes, little is known regarding the conductive properties of the newly formed surrounding tissue. In particular, it is not clear how the ventral side of the connective tissue between the brain and the electrode array affects the quality of signals. Some studies have reported that encapsulation with both dural thickening and newly formed fibrous tissue may dislodge the implants (Schendel et al., 2013, 2014). On the ventral side, the newly formed tissue thickened the distance between the signal source and electrodes and increased the 1-kHz-electrical impedance (Henle et al., 2011; Mestais et al., 2015). This would reduce the quality of the signal recording. The correlation between long-term tissue reactions and neural signal quality is not determined yet (Degenhart et al., 2016; Kaiju et al., 2017).

However, the functional results of long-term recordings and histological evaluations in such systems are still limited, and further research is required to facilitate the optimization of device design and manufacturing. In this study, we explored both host-tissue response and recording function of a customized wireless ECoG device after 15 months of its implantation. We evaluated cortical tissue changes and fibrosis at the implant site and the device performance by auditory steady-state response (ASSR) testing and spectrogram analysis. We also assessed the signal stability by comparing the root mean square (RMS) voltage and power spectral density (PSD) results in the acute and chronic phases.

## 2. Materials and methods

### 2.1. Ethics approval and consent to participate

The Institutional Review Boards approved this study for animal experiments at both Astellas Pharma Inc. (D-T20002-01) and Osaka University (02-007-000). All experiments in this study were performed at the Tsukuba Research Center of Astellas Pharma Inc., which is accredited by AAALAC International. Studies involving animals are reported in accordance with the ARRIVE guidelines (Percie du Sert et al., 2020).

### 2.2. Wireless implantable device

**Figure 1:**
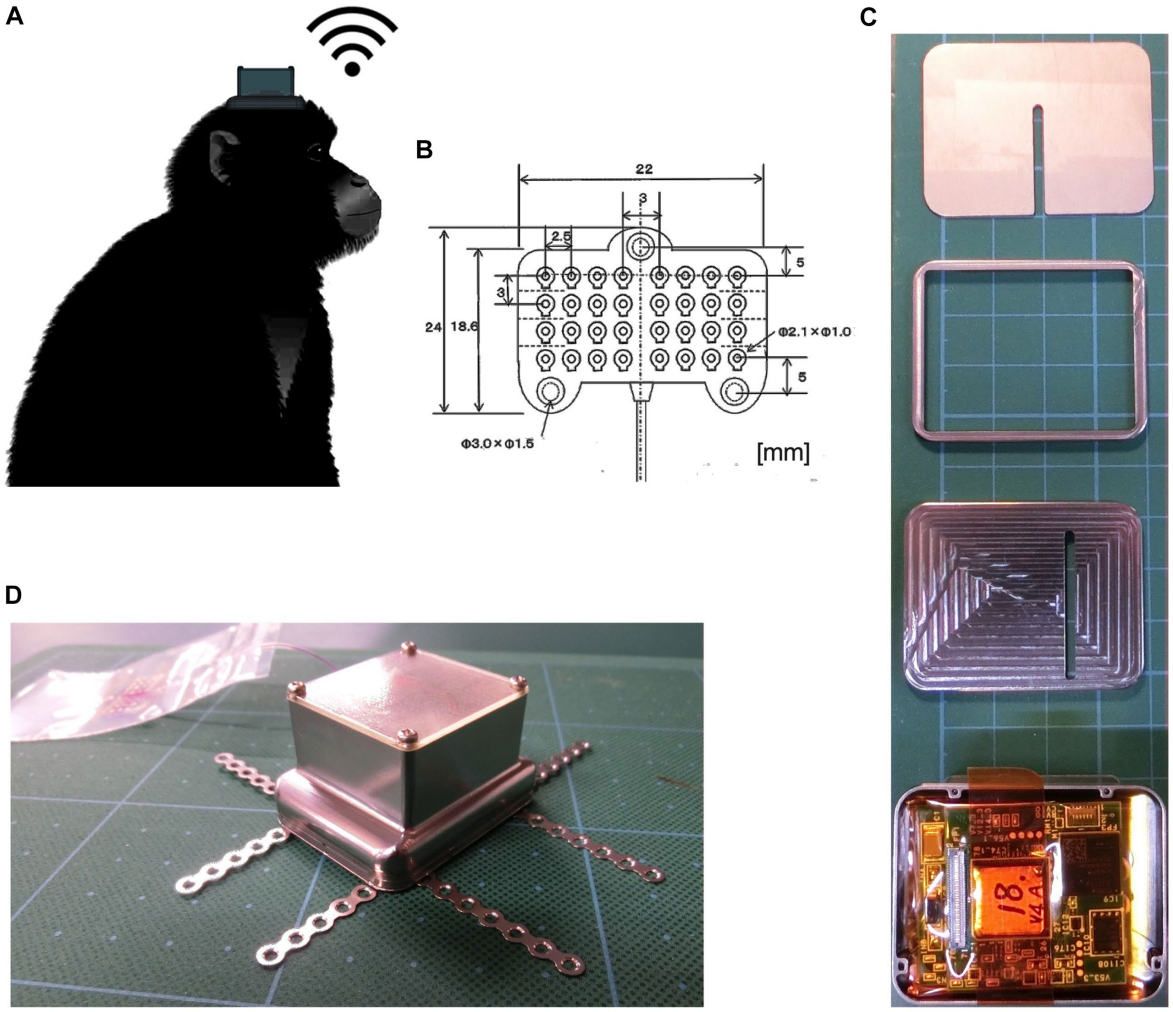
Photographs of the implantable wireless 32-ch ECoG device. **(A)** Monkey with a wireless recording device implanted on top of the skull. **(B)** The 32-channel silicone-based subdural electrode array, with a thickness of 0.7 mm. **(C)** Measurement units (from top to bottom: skull side surface, connector part, intermediate layer separating measurement and power units, a 32-ch amplifier AFE chip and a 2.4-GHz wireless data transfer module). **(D)** A photograph of the device ready for implantation.

We used a customized wireless implantable ECoG recording device in non-human primates (Figure 1A). The device consisted of a subdural silicon-based 4 × 8 electrode array and a wireless recording unit (Figure 1B). The recording electrodes were 1.0 mm in diameter, with an interelectrode spacing of 3.0 or 2.5 mm to ensure wide cortical coverage. The wireless recording unit consisted of microelectronic, wireless telemetry components and batteries assembled within a titanium casing fixed on the skull by a screw (Figures 1C,D). The bottom section of the casing contained an analog front-end (AFE) chip (Yoshida et al., 2011) to amplify 32-ch signals with peak-to-peak noise less than 6 μV, to perform bandpass filtering, and to convert analog voltage signals to 14-bit digital data at a sampling rate of 1,000 Hz. The data management unit transfers ECoG data at the maximum effective speed of 2.0 Mbps from the measurement units to the external receiver. In the external receiving system, we used an in-house data monitoring software package to monitor real-time data recording. The software can receive and display 32-ch signals, and enable adjustment of some parameter settings, such as gain, bandpass filter.

### 2.3. Implantation and electrocorticography recording

To record brain signals, we implanted the devices in two 6-year-old male cynomolgus monkeys (weighing 6.8 and 6.9 kg, respectively, obtained from Shin Nippon Biomedical Laboratories Ltd., Tokyo, Japan). After general isoflurane (2–4%), a J-shaped skin incision was made on the midline from the nasion to the inion and then curved to the left ear lobe. Bleeding was carefully coagulated using a bipolar coagulator. Then, a 30 × 20 mm square craniotomy was performed on the left temporoparietal bone under microscope. A 10 × 20 mm horseshoe-shaped dural membrane flap was cut and folded to expose the subdural area. Our electrode array was placed over left auditory and somatosensory cortex. Finally, the dural membrane was sutured water-tight, with a layer of artificial dural membrane (Neoveil, Gunze, Japan) and adjuvant sealant hydrogel (Adherus, Striker, Tokyo, Japan). The bone flap was fixed with two sets of titanium plates and screws. The device casing was fixed to the middle line on frontoparietal skull by using titanium screws. The dead space between the skull and the device was filled with dental cement (GC Corporation, Tokyo, Japan) to prevent infection. We left the top section of casing exposed, because the assembly was too bulky to allow complete skin closure.

Two weeks after the surgery (acute phase), we subjected the monkeys to ASSR followed by Ketamine tests on two different consecutive days. For ASSR testing, the monkeys were individually positioned in primate chairs (O’HARA & CO., LTD., Tokyo, Japan) for signal recording. The antennas for wireless recordings were placed 1.5 m in front of the monkey. The monkeys were then exposed to auditory stimuli via two loudspeakers, which consisted of click sounds presented with a 500-ms duration of 40-Hz trains at 1,100-ms intertrain intervals and repeated 200 times/trial. In the Ketamine tests, the monkeys were placed in a cage allowing free movement. Spontaneous ECoG signals were monitored for 60 min to obtain baseline data. After this baseline, intramuscular (im) injections of ketamine were administered at a dose of 3.5 mg/kg. The monkeys were then placed back into the cage allowing for free moving with spontaneous ECoG monitor for 60 min after ketamine injection. After 15 months of the implantation (chronic phase), we performed ASSR and Ketamine tests again using the same procedure to compare the functional performance and complete the electrophysiological experiments.

### 2.4. Signal processing

Raw ECoG data were analyzed offline using MATLAB (MathWorks Inc., R2016a, Natick, MA, United States). Raw data were bandpass filtered at 0.1–200 Hz. For the ASSR analysis, ECoG data were segmented into 1,100-ms epochs concurrent with the train stimulus. ASSRs averaged over 200 epochs were analyzed. Spectrogram analyses were performed using a 256-point fast Fourier transform (FFT; baseline: −100–50 ms) with a Hamming window (10%). For the time-frequency analysis of ketamine administration, waveforms were transformed to the frequency domain using an FFT and a Hamming window with a 50% overlap between data blocks and a block size of 1,024. The spectrograms were then averaged in 1-min bins to develop a heat map.

### 2.5. Measurement of the ventral tissue gain

To measure the transfer function of the newly formed tissue between the electrode array and cortex, we used a previously published method (Torres Valderrama et al., 2010). In general, assuming that the tissue behaves as a linear, time-invariant system characterized by a gain function $G\left(f\right)$ , the power spectral density after 2 weeks of implantation $P_{bef}\left(f\right)$ , is related to that after 15 months $P_{\mathrm{aft}}\left(f\right)$ , by $P_{\mathrm{aft}}\left(f\right)=G\left(f\right)P_{bef}\left(f\right)$ . Under the assumption of stationarity, the estimate of the gain function $\hat{G}\left(f\right)$ isgiven by

$$\hat{G}\left(f\right)=\frac{{\hat{P}}_{\mathrm{aft}}\left(f\right)}{{\hat{P}}_{bef}\left(f\right)}\text{,}$$

where ${\hat{P}}_{bef}\left(f\right)$ and ${\hat{P}}_{\mathrm{aft}}\left(f\right)$ are the estimates of the power spectral density after acute and chronic implantation, respectively. The presence of the newly formed tissue between the brain surface and array is represented by the frequency-dependent gain factor $\hat{G}\left(f\right)$ . Non-functional electrodes were excluded from this analysis.

### 2.6. Measurement of the power spectral density

To compute the power spectra, FFT with Hamming windows was applied to the signal. The μV^2^ power values were calculated using the pop_spectopo.m function in EEGLAB (Delorme and Makeig, 2004) with Welch’s power spectral density estimate (5-s window length, 80% overlap; Delorme and Makeig, 2004). The spectrogram of the mean PSD across the electrode channels and animals was then computed and averaged in 1-min bins to develop a heatmap of the spectrogram.

### 2.7. Measurement of the root mean square

To assess signal stability, we calculated the root mean square (RMS) voltage for 60-min recordings after ketamine injections. RMS voltage is a widespread characteristic that represents the average voltage level over a certain period (Larzabal et al., 2021). The average RMS signal was computed in the 0.1–200 Hz frequency range in both acute and chronic phases.

### 2.8. Explantation and immunohistochemistry

Fifteen months after implantation and completion of electrophysiology experiments, both monkeys were euthanized, and their entire bodies were perfused transcardially with 10% formalin. After fixation, the implanted device was carefully removed under a microscope. To avoid damaging the brain tissue, we extracted the grid electrodes using a surgical microscope. The entire brain, dura, and surrounding tissue were then fixed in 10% formalin and embedded in paraffin for further histological evaluation.

We coronally sectioned four slices of the cortical and surrounding tissues from both the implanted (left) and non-implanted control (right) sides of the brain for comparison. All slides were processed under the same conditions to minimize operational errors. Sections were processed for Nissl staining and immunohistochemical staining of neuronal nuclei (NeuN, 1:1,000, Millipore), glial fibrillary acidic protein (GFAP, 1:500, Diagnostic BioSystems), ionized calcium-binding adapter molecule 1 (Iba-1, 1:500, GeneTex), and vimentin (Vim, 1:500, Leica). The tissues were first blocked for 10 min in sodium citrate buffer (0.1 M citric acid, 0.1 M sodium citrate, pH 6.0) at 121°C. After inactivation of endogenous peroxidase with 3% H_2_O_2_ in methanol for 15 min at room temperature, the tissues were incubated with primary antibodies overnight at 4°C. Following washes in phosphate buffer saline (0.05 M PBS, pH 7.6), the tissue was probed with anti-mouse IgG antibody labeled with peroxidase secondary antibody (Histofine Simple Stain MAX PO; Nichirei, Jp) for 30 min at room temperature. The sections were visualized using 3,3′-diaminobenzidine tetrahydrochloride (Nichirei) at room temperature. The sections were subsequently counterstained with Mayer’s hematoxylin and examined under a microscope. Images of the sections were captured using a microscope (BZ-X800, Keyence, Japan) at 4× and 20× magnification, manually outlined, and quantitatively measured using BZ-X800 software.

The encapsulating tissues from the ventral and dorsal sites, and the control dural membrane, were identified under the microscope and the tissue thicknesses were determined by averaging 10 sampling points on each section (in total, *n* = 40 per group). Comparisons between the ventral and dorsal encapsulations were performed using paired t-test.

### 2.9. Statistics

Prism v9.0 (GraphPad Software Inc., La Jolla CA) was used for all statistical analyses. The data are shown as mean ± SD, and the level of statistical significance is set at *p* < 0.05.

## 3. Results

### 3.1. Signal quality

**Figure 2:**
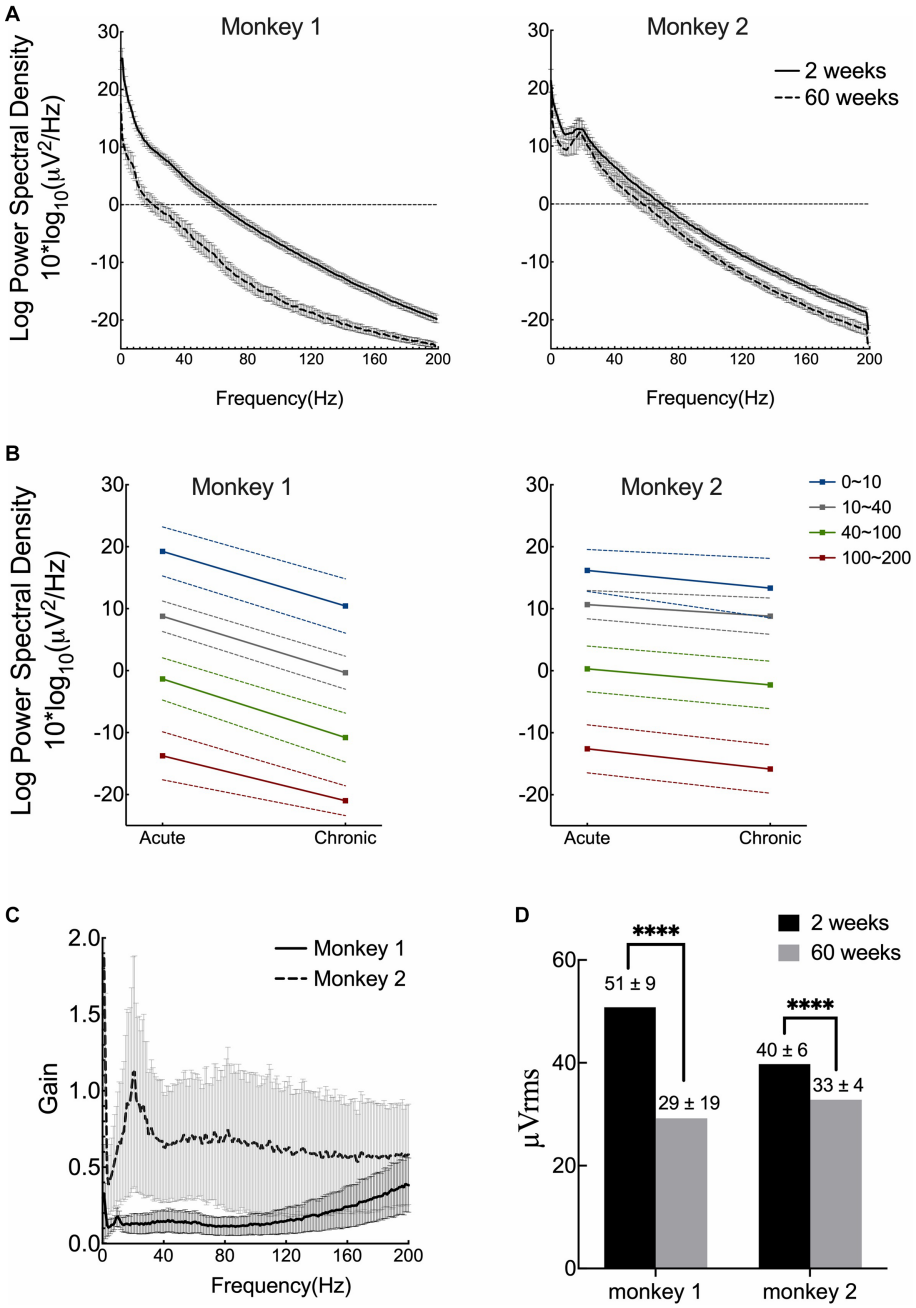
Signal quality and stability over 15 months. **(A)** Mean results of power spectral density (PSD) of both acute and chronic phases for two monkeys. **(B)** Median power spectral density with standard deviation across four frequency bands. **(C)** Estimate of the gain of tissue proliferation on the ventral side *in vivo*. The lines correspond to the mean results computed from 31 channels for each monkey (the channel with poor recording was excluded). **(D)** Comparison of the root-mean square (RMS) voltage in the acute and chronic phases in both monkeys. (Acute phase: 2 weeks after surgery; chronic phase: 15 months after implantation).

To estimate the signal quality, we plotted the mean RMS, PSD, and gain for both monkeys and compared the results between the acute and chronic phases. PSD was computed over an hour after ketamine administration. As shown in Figures 2A,B, the frequency-dependent PSD and median signal power decreased as the frequency increased. After 15 months of implantation, the PSD decreased over all frequency bands in both monkeys, and was more evident in monkey 1. The estimate of the gain of the newly formed ventral tissue was computed based on the signals recorded during the acute and chronic phases (Figure 2C). The curve represents the averaged results for 31 channels (the channel with poor recordings was excluded from this analysis). The results showed that tissue proliferation between the brain surface and electrodes reduced the amplitude (power) of ECoG signals. The effect in monkey 1 was dramatically greater than that in monkey 2, 15 months after implantation. The average RMS voltages (shown in Figure 2D) for monkey 1 remained at 60% from 50.8 μV (SD = 9.4) to 29.2 μV (SD = 19.2). While for monkey 2, the value remained at approximately 80% from 39.8 μV (SD = 5.7) to 32.8 μV (SD = 3.5). In summary, the implants saw a roughly 20–40% reduction in their RMS after 15 months.

### 3.2. Time-frequency analysis

**Figure 3:**
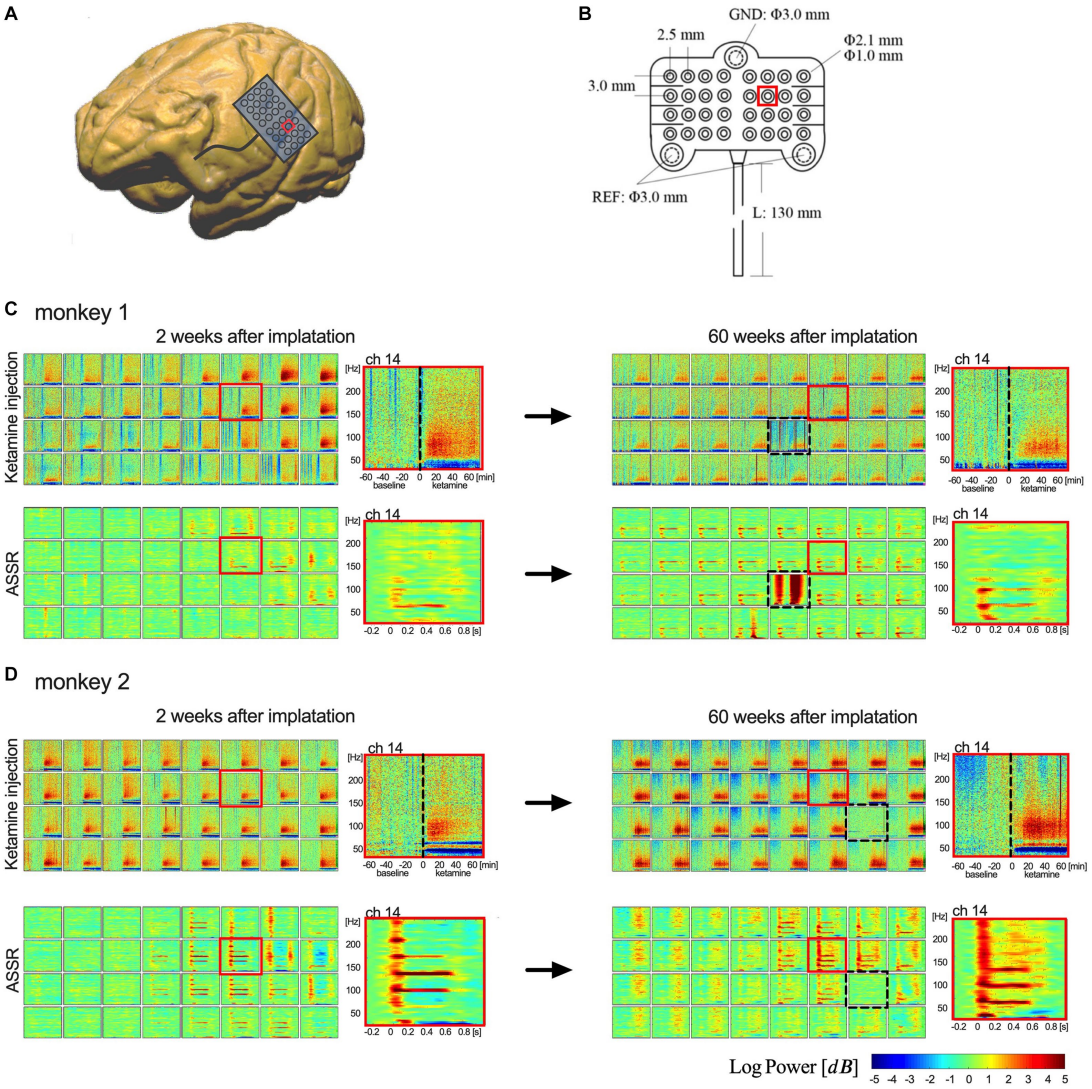
Implanted electrodes array and comparison of the recording performance. **(A,B)** Show the 32-channel array and its implantation area on brain surface (red square represents location of channel 14). **(C,D)** Show the time-frequency analysis of ketamine tests and ASSR between 2 weeks (left column) and 60 weeks (right column). The upper rows of spectrograms in **(C,D)** show the results of ketamine test (one-hour-baseline and one-hour-recording after ketamine); the lower rows show the results of average 40-Hz ASSR between −0.2 and 0.8 s. Each box represents an electrode in the 4 × 8 array. 32 channels are shown in an order from upper left (ch 1) to lower right (ch 32). [Dotted boxes in **(C,D)** indicate malfunctioning channels. Red boxes represent locations of channel 14].

To test the long-term recording performance, we compared the time-frequency analysis of ASSR and ketamine tests between their acute and chronic phases for both monkeys. The results for each monkey are shown separately in Figure 3. After 15 months from the implantation, raw ECoG signals were recorded and transmitted from most electrodes (62 of 64 in total). For each monkey, the signals recorded from one electrode indicated malfunctioning. In monkey 1, channel 21 showed abnormal responses, and in monkey 2, channel 23 failed to manifest a clear output. All other electrodes demonstrate adequate function to detect cortical signals. Time-frequency spectrogram analysis showed typical ASSR responses at 40 Hz with 80/120 Hz harmony echoes and a characteristic increase in broadband gamma (>30 Hz) activity after ketamine injection. Similar results were observed in the acute and chronic phases in both monkeys. These combined results demonstrate this wireless neural interface to perform well during chronic ECoG recording in nearly all electrodes over a 15-month period.

### 3.3. Dural reactions

**Figure 4:**
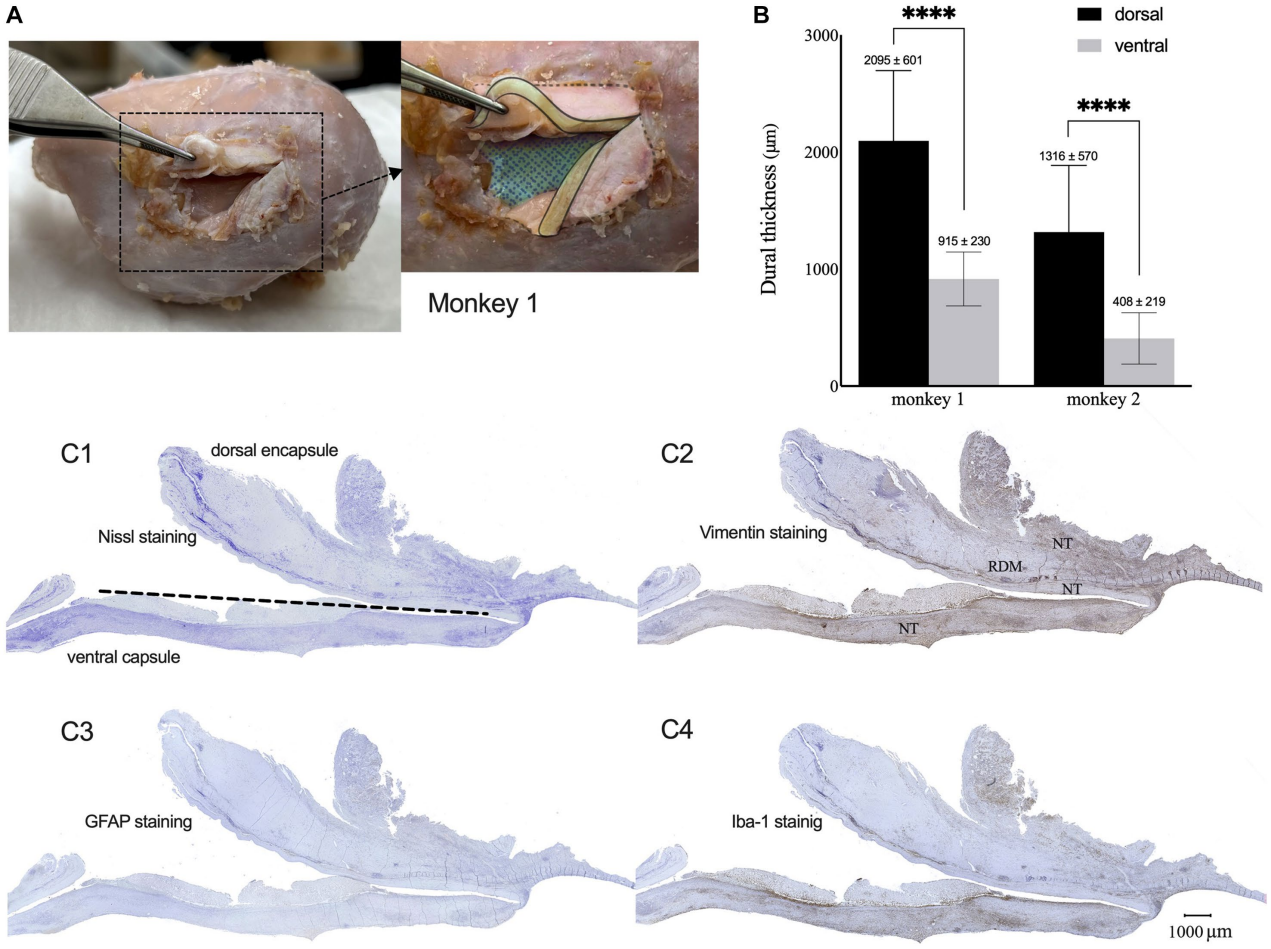
Dura reactions over 15 months of the implantation. **(A)** Macroscopic observation of tissue encapsulation on the implanted site for monkey 1 (cortical surface was colored with blue mesh, sections of thickened dura were colored with yellow). **(B)** Mean thickness of the dorsal and ventral encapsulation for both monkeys. **(C1–C4)** Representative Nissl **(C1)**, Vim **(C2)**, GFAP **(C3)**, and Iba-1 **(C4)** expression patterns in the capsulation from monkey 1. (NT: newformed tissue; RDM: reactive dural membrane).

Throughout the implantation period, we did not observe any adverse effects or abnormal symptomatic motor behavior in either monkey. After sacrifice, we did not observe any macroscopic signs of tissue defects, except for thickened connective tissue formation in the dural membrane and encapsulation of the electrode array. However, the electrode array was easily extracted from the encapsulating tissue. The proliferated fibrous tissue tightly adhered to the dural membrane (Figure 4A). The brain parenchyma underneath the encapsulated electrodes was mechanically depressed.

Nissl staining of the surrounding tissue revealed fibrous proliferation on both epidural and subdural sides of the dura mater. Dorsal encapsulation included the newly formed tissue (NT) and reactive dura mater (RDM), and gradually became thicker from the edge toward the center, while its thickness was much greater than that of ventral encapsulation (only newly formed tissue). The average thickness of the dorsal encapsulation (1,760 ± 701 μm) was significantly greater than that on the ventral side (661 ± 339 μm, *t*-test, *p* < 0.01; Figure 4B).

Immunohistochemical examination showed GFAP, Iba-1, and Vim expression patterns in the surrounding tissue (Figures 4C1–C4 for monkey 1, Supplementary Figure 1 for monkey 2). GFAP-positive astrocytes were not observed, indicating the absence of astrogliosis in the encapsulation. We found Iba-1-labeled macrophages and Vim-labeled fibroblasts with increased densities in the NT-RDM and NT-array borders and the outer layers of RDM and NT. These results indicated that the accumulation of inflammatory macrophages and meningeal-derived fibroblasts resulted in newly formed connective tissue in the subdural spaces between the electrode array, cortex surface, and dural membrane.

### 3.4. Brain tissue reactions

**Figure 5:**
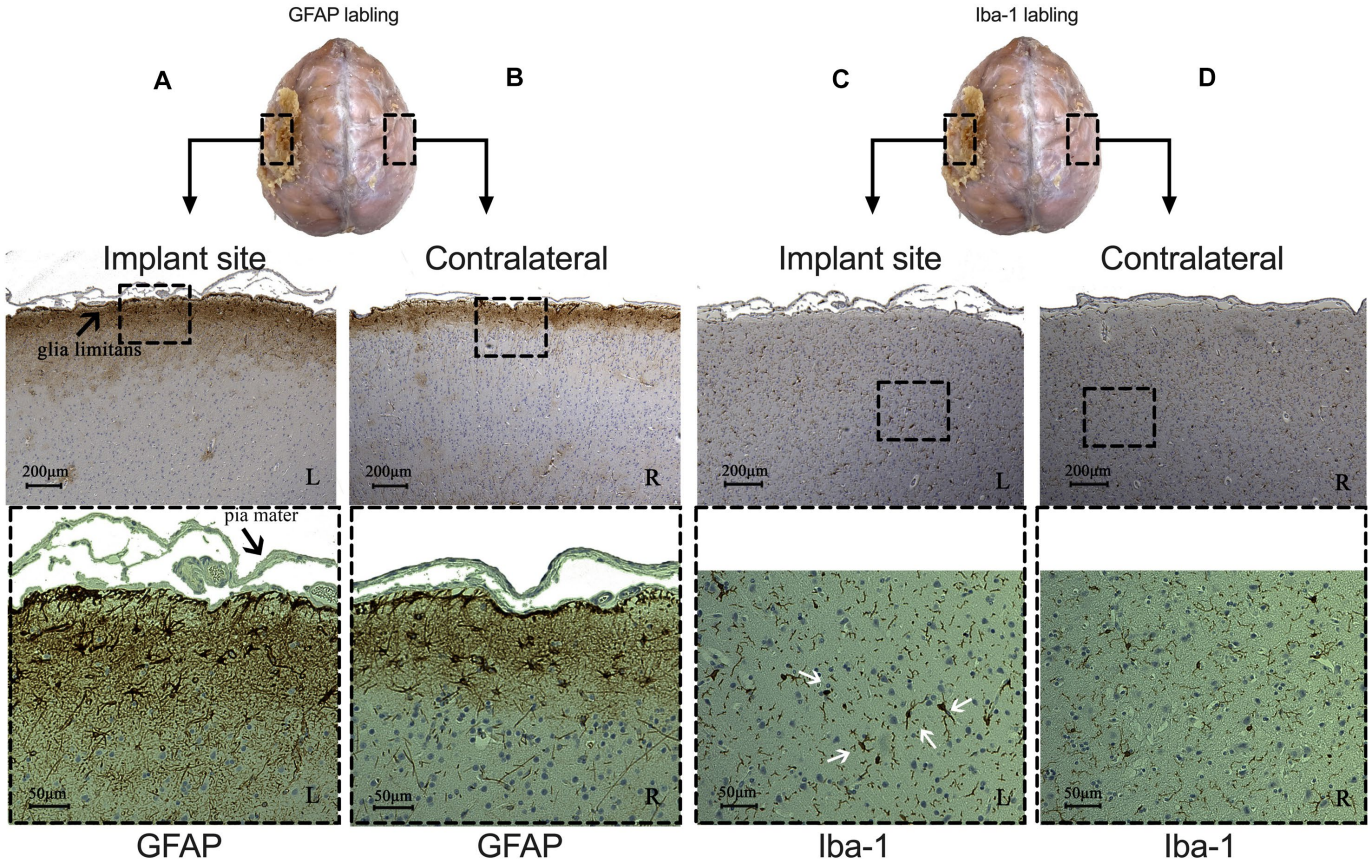
Comparison of immunohistochemical results between implant (left side) and contralateral sites (right side) from monkey 1. **(A,B)** Signal of astrocytes labeled with GFAP under implant **(A)** and contralateral site **(B)**. CD: Microglia labeled with Iba-1 under implant **(C)** and contralateral site **(D)**. (White arrows indicate reactive microglial cells. L: left, implant site: R: right, control site).

We then performed brain tissue immunohistochemistry to evaluate cortical cytoarchitecture reactions at the implanted sites to detect signs of chronic inflammation. We did not observe abnormal neuronal morphology from Nissl or NeuN staining on either side. Results from monkey 1 were shown in Figure 5 (we showed monkey 2 in Supplementary Figure 2). The signal of GFAP-labeled astrocytes was highly increased in the glia limitans and in layer I of the brain cortex (Figure 5A), compared to the deeper layers and the contralateral side (Figure 5B). Astrogliosis in glia limitans increased its thickness, and we did not observe any loss of continuity for the glia limitans. Iba-1-labeled immunohistochemistry showed the presence of reactive microglial cells, with the appearance of a large, round cellular body and short, thick, or retracted branches (Figure 5C). On the contralateral side, resting microglia are composed of long-branching processes and a small cellular body (Figure 5D). We did not observe similar concentration of microglia in glia limitans on the contralateral, control hemisphere. These results indicate that the electrode array induced a mild brain tissue response only to the most superficial cortical layers over a 15-month period.

## 4. Discussion

In this study, we aimed to validate the long-term biocompatibility of the implanted electrode array and casing, and assess the quality of the ECoG signal after 15 months of implantation. Previously, we published evaluation results of the electrode array and device in the acute phase (Yan et al., 2020, 2022). As a follow-up analysis of the device, we computed the average RMS and PSD of the signal, plotted the time-frequency spectrogram of the raw data, examined the post-mortem histology results, and compared the results with those of the acute phase. In addition, we first studied the effects of the formation of connective tissue between the brain surface and the electrode array on the quality of ECoG signals. Our results demonstrate the relative stability of the signal and less compatibility of the device due to a foreign body reaction in two monkeys after 15 months of the implantation.

### 4.1. Long-term biocompatibility

We observed typical fibrotic growth encapsulating the electrode array, with shallow mechanical depression of the brain parenchyma after 15 months. A previous study suggested that the thickened reactive tissue merely induced superficial compressive deformity by subdural implants but did not affect the normal layering structure of the mildly compressed brain tissue (Degenhart et al., 2016). Over the chronic phase, the brain was able to accomodate the presence of the new tissue without detectable alteration in function. Fibrous connective tissue was observed at both the dorsal and ventral of the electrodes. Microscopic observation showed that the accumulation of inflammatory macrophages and meningeal-derived fibroblasts led to newly formed connective tissue in the subdural space, resulting in the proliferation of the dural membrane with newly formed tissues in the epidural space between the membrane and skull. This is similar to previous reports that progressive fibrous overgrowth completely encapsulated electrodes as early as 1 month after implantation (Schendel et al., 2013, 2014; Degenhart et al., 2016). Tissue encapsulation is the final stage of anti-inflammatory wound healing and persists chronically throughout the lifetime of the implant (Anderson et al., 2008; Lynn et al., 2011). We also observed a gradient where the surrounding tissue more closely represented the newly formed fibrous tissue as a foreign body response, and reactive dura mater thickening with the newly formed tissue on the epidural side more closely as a traumatic reaction of durotomy and craniotomy. As there were only leptomeninges separating the brain and array at the time of implantation, it is assumed that the ventral encapsulation grew *de novo* post-implantation (Degenhart et al., 2016; Romanelli et al., 2018). Therefore, further effort should be focused on reducing ventral encapsulation.

We then evaluated brain tissue to detect signs of inflammation. We observed an increase in astrogliosis in layer I and limitans compared to the contralateral side. This expression pattern is considered a native immune response to trauma or chronic foreign body implantation to establish a physical and immunological barrier (Peters and Sethares, 2002; Griffith and Humphrey, 2006). Microglial activation was observed under the electrode array, with no aggregation in limitans nor movement to the peripheral area. This indicated that microglial changes were residual after operative damage and not actively responding to subdural implants (Kozai et al., 2012; Roth et al., 2014; Degenhart et al., 2016). Overall, the devices were well-tolerated for 15 months.

### 4.2. Electrocorticography recording quality

We have shown the time-frequency spectrogram results of the 40-Hz ASSR, and ketamine-induced power increase in the broad gamma and high-gamma bands (Yan et al., 2022). These two well-established neuroscientific biomarkers were used to evaluate functionality of the device and to show its capacity to detect signals. The 40-Hz ASSR can be used to interpret and differentiate the neural reaction to sound stimuli at millisecond precision. Acute ketamine administration induces a state of high neuronal excitability and thus increases broadband gamma activity. In this chronic experiment, we demonstrated similar results to those obtained in the acute phase. The signal recording and data transmission functions performed well after 15 months of implantation. In each array with 32 electrodes, only one failed to show good recording capability.

PSD is commonly used to quantitatively assess the power of each frequency in ECoG recordings. Generally, ECoG signal amplitudes decrease as the frequency increases, which is characteristic of mammalian signals (Buzsaki and Draguhn, 2004). RMS voltage is a widely used index for assessing the stability of an ECoG signal. We found a decrease in PSD and RMS values for both monkeys after 15 months of implantation, which can be attributed to tissue formation on the ventral side. This is similar to previous reports showing that PSD is higher in subdural recordings than in epidural (Bundy et al., 2014; Sun et al., 2018), and the RMS voltages showed decrease over 1 or 2 years (Ryapolova-Webb et al., 2014; Swann et al., 2018; Larzabal et al., 2021).

We then computed the gain of the newly formed tissue and demonstrated that the attenuated amplitude of the ECoG signals is possible because of the presence of the tissue between the brain surface and the electrode array. This result is similar to that of a previous study (Torres Valderrama et al., 2010), which studied the normal human dura mater. However, the reactive tissue is much thicker than the normal dura mater, and the differences in PSD, RMS, and gain between the two monkeys are assumed to originate from the thicker tissue (ventral side) in monkey 1. We did not directly apply electrical signals to the dura mater. The estimate of the gain depended on neural activity signals, which are susceptible to ketamine injection. Although long-term tissue reactions decreased the amplitude of ECoG signals, its effect was limited. In terms of signal quality, the analysis and performance of the power spectral features remained similar in both monkeys.

### 4.3. Chronic failure mode analysis

Several factors can lead to the failure of BMI implants. Chronic factors can be broadly subdivided into biological, material, and mechanical failures (Prasad et al., 2012; Takmakov et al., 2015; Delbeke et al., 2020). Biological failures are defined as those related to inflammatory reactive tissue responses to implanted electrodes (Karumbaiah et al., 2012; Kozai et al., 2014). Encapsulation of meningeal tissue and fibrogenesis can increase the distance between foreign bodies and the brain surface, leading to sensor failure (Polikov et al., 2005; Prasad et al., 2012). Approximately 24% of the failures are chronic biological failures (Barrese et al., 2013). Material failures are related to the material degradation of the connector (Simeral et al., 2011; Barrese et al., 2013), decomposition or delamination of insulation (Barrese et al., 2013), corrosion of metallic electrodes (Prasad et al., 2012), crack propagation, and ionic contamination (Prasad et al., 2014; Sankar et al., 2014; Kozai et al., 2015a,b). Mechanical failures are related to physical factors that eliminate an electrode’s conductive path from the sensor recording site to the signal processors, such as breakage of the cable or loss of polymeric insulation (Moxon et al., 2009; Prasad et al., 2012). In chronic implants, the host response at the tissue-electrode interface eventually leads to mechanical failure and signal degradation (Campbell and Wu, 2018).

Our data showed that it was feasible to record useful signals from the device for more than 1 year, but the recording quality, number of channels, and signal amplitude diminished over long periods. The post-explanation examination of the device did not show failures on the electrodes, silicone array, or cables. The aforementioned histological analysis did not reveal any biological failure. The major chronic problem was supposed to be a biomechanical factor from the grossly observed meningeal encapsulation that distanced the electrode array from the brain surface. This is a major contributing factor to the reduction in the power and signal quality over a long period.

### 4.4. Comparison to epidural implants

Considering lower invasiveness, some studies used epidural electrode arrays such as WIMAGINE (Sauter-Starace et al., 2019). Different from subdural implantation, epidural electrodes were implanted in the space between dura mater and skull. Because of the separation by dura mater, the electrode array exerted less effect on brain tissue. Without durotomy and water-tight sutures, it was easier and safer to perform implantation surgery. However, because of a larger distance between electrode and brain surfaces, PSD results from epidural implants were obviously lower than that from subdural electrodes. Especially for high gamma activity, higher signal power is required for BMI applications.

### 4.5. Implications

When designing the subdural ECoG device, a key element could alleviate inflammatory reactions at the electrode-tissue interface, especially ventral encapsulation. Fibroblasts play a critical role in this “structural immunity” response to tissue injury. They initiate inflammation in the early stages by expressing chemokine synthesis and regulation of hematopoietic cells. Immune cells then respond and provoke a cascade of events to clear the invasive microorganisms and form the collagenous envelope (Krausgruber et al., 2020; Armingol et al., 2021). To reduce local inflammation and electrode degradation while maintaining electrical sensitivity, multiple strategies such as altering the shape of the array substrate, increasing array flexibility, anti-fouling coating of the array substrate, and releasing anti-inflammatory drugs from the array substrate or electrodes (Degenhart et al., 2016; Gulino et al., 2019), have been suggested.

### 4.6. Limitations

This study has several limitations. There is no impedance measuring instrument in our device; therefore, we could not measure the contact impedance during the experiments. Several studies have continuously measured impedance over a long period and found a close relationship with chronic inflammatory reactions. Signal amplitude attenuation should correlate with impedance change (Schendel et al., 2014; Larzabal et al., 2021). In addition, we only tested the device in two phases, after 2 weeks and after 15 months. The lack of continuous measurements made it impossible to investigate the detailed changes we observed between the acute and chronic phases. In this study, we were not able to close the scalp because of the size and bulk of the titanium casing. Despite our use of dental cement to minimize dead space, the lack of complete closure increased the risk of infection and may have enhanced the inflammatory responses that led to the tissue thickening that we observed. Future designs should aim to test fully implantable devices with wireless charging technology to determine if fully enclosed devices might be associated with reduced thickening of encapsulating tissue in the chronic recording phase. Lastly, our study is limited by a small sample size, with more studies needed to confirm our conclusions. We believe that our results provide useful arguments on the chronic host response, long-term functionality of the ECoG device, and characterization of the influence of the ventral connective tissue on signal detection over a long period of time.”

## 5. Conclusion

Fifteen months after implantation, we evaluated the functionality and biocompatibility of the wireless ECoG recording device in two awake monkey models. The mean RMS and PSD results showed decrease after 15 months, and time-frequency analysis of ASSR and ketamine experiments showed similar results for signal feature detection compared to the acute phase. A post-mortem examination showed thickening of the reactive tissue around the electrode array, but no evident inflammation in the cortex. In addition, for the first time, we calculated the attenuation (gain factor) of the ECoG signals by ventral tissue proliferation *in vivo*. We suggest that reducing the thickness of the ventral tissue would benefit subdural signal recording performance. The outcome of this preclinical study confirms its ability to record neural activity through fibrous proliferation and transmits data wirelessly through the scalp over a long-term period. This represents a major step toward future clinical trials and neuroscientific studies.

## Data availability statement

The raw data supporting the conclusions of this article will be made available by the authors, without undue reservation.

## Ethics statement

The animal studies were approved by アステラス製薬㈱つくば研究センター動物実験委員会 (D-T20002-01) and 大阪大学大学院医学系研究科動物実験委員会 (02-007-000). The studies were conducted in accordance with the local legislation and institutional requirements. Written informed consent was obtained from the owners for the participation of their animals in this study.

## Author contributions

TY: Data curation, Formal analysis, Investigation, Software, Validation, Visualization, Writing – original draft. KS: Formal analysis, Investigation, Methodology, Software, Validation, Writing – review & editing, Data curation. SK: Data curation, Formal analysis, Investigation, Methodology, Resources, Software, Visualization, Writing – review & editing. MM: Data curation, Investigation, Methodology, Project administration, Resources, Writing – review & editing. TM: Writing – review & editing. MH: Conceptualization, Data curation, Funding acquisition, Investigation, Methodology, Project administration, Resources, Supervision, Writing – review & editing.

## Funding

The author(s) declare financial support was received for the research, authorship, and/or publication of this article. This study received support from the joint research fund between Osaka University and Astellas Pharma Inc. and the joint research fund between Nihon Kohden Corporation, Murata Manufacturing Co. Ltd., JiMED Co. Ltd., Osaka University, JST SPRING (Grant number JPMJSP2138), JSPS KAKENHI (Grant number 21H03312), and a commissioned research (No. 22801) by National Institute of Information and Communications Technology (NICT), Japan.

## Conflict of interest

MH is the representative researcher of the joint research fund between Osaka University and Astellas Pharma Inc. and the joint research fund between Nihon Kohden Corporation, Murata Manufacturing Co. Ltd., JiMED Co. Ltd., and Osaka University. MH owns stock of the start-up company JiMED.

The remaining authors declare that the research was conducted in the absence of any commercial or financial relationships that could be construed as a potential conflict of interest.

## Publisher’s note

All claims expressed in this article are solely those of the authors and do not necessarily represent those of their affiliated organizations, or those of the publisher, the editors and the reviewers. Any product that may be evaluated in this article, or claim that may be made by its manufacturer, is not guaranteed or endorsed by the publisher.
