# Supplementary material for: Chronic subdural electrocorticography in nonhuman primates by an implantable wireless device for brain-machine interfaces
Source: Front Neurosci. 2023 Sep 28;17:1260675. doi: 10.3389/fnins.2023.1260675 (PMC10568031; doi:10.3389/fnins.2023.1260675)
Supplement: Supplementary file 1 [file Data_Sheet_1.PDF]

Supplementary Material

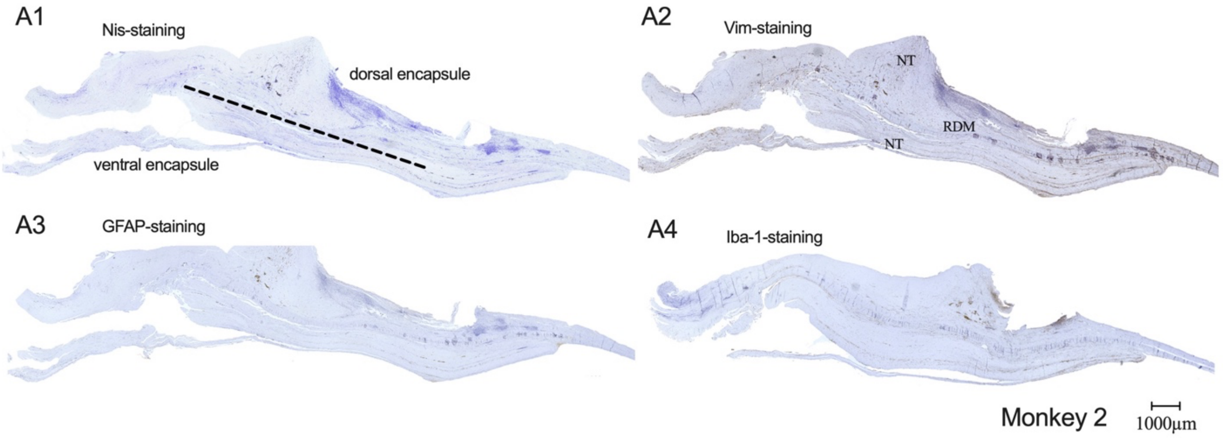

Supplementary Figure 1. Dura reactions over 15 months of the implantation for monkey 2. A1: Nissl staining; A2: Vimentin staining; A3: GFAP staining A4: Iba-1 staining. (NT: newformed tissue; RDM: reactive dura membrane).

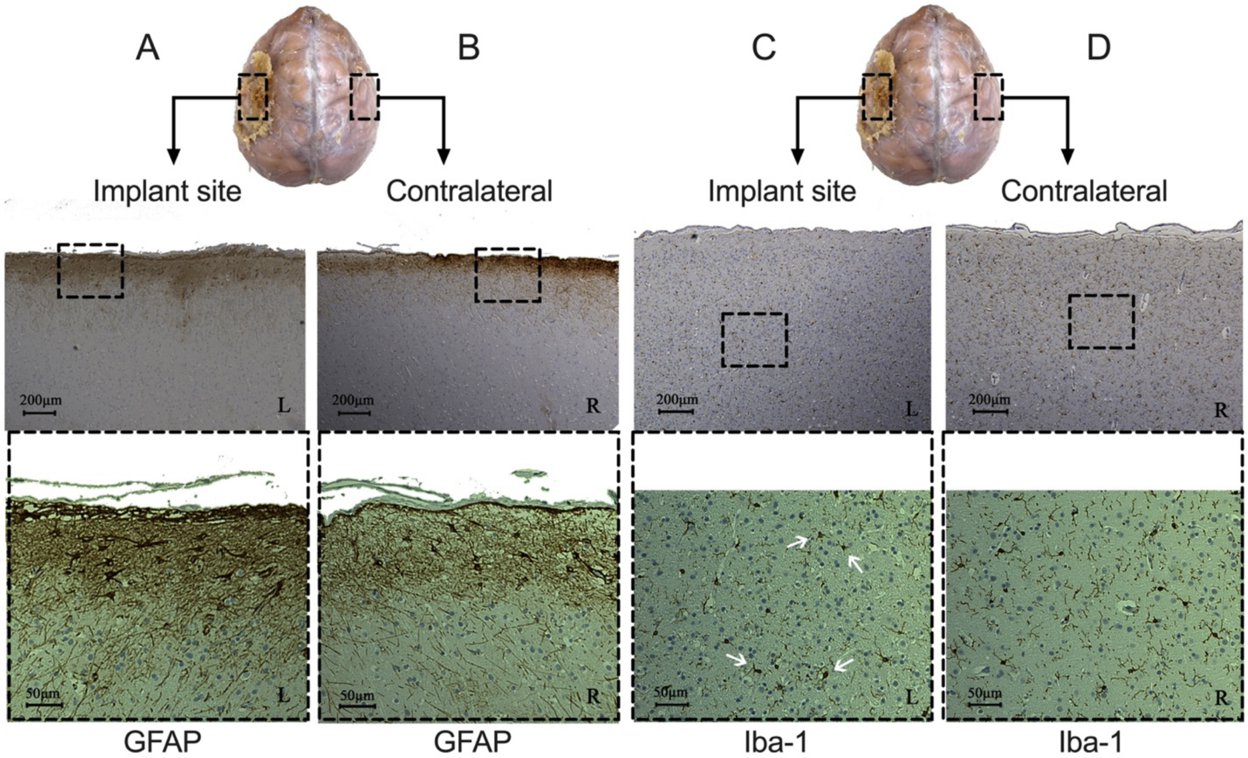

Supplementary Figure 2. Comparison of immunohistochemical results between implant (left side) and contralateral sites (right side) from monkey 2. A and B: Signal of astrocytes labeled with GFAP under implant (A) and contralateral site (B). C and D: Microglia labeled with Iba-1 under implant (C) and contralateral site (D). (White arrows indicate reactive microglial cells. L: left, implant site: R: right, control site).
